# Impact of cardiac output and alveolar ventilation in estimating ventilation/perfusion mismatch in ARDS using electrical impedance tomography

**DOI:** 10.1186/s13054-023-04467-w

**Published:** 2023-05-08

**Authors:** Samuel Tuffet, Tommaso Maraffi, Matthieu Lacheny, François Perier, Anne-Fleur Haudebourg, Mohamed Ahmed Boujelben, Glasiele Alcala, Armand Mekontso-Dessap, Guillaume Carteaux

**Affiliations:** 1 CHU Henri Mondor-Albert Chenevier, Service de Médecine Intensive Réanimation, Assistance Publique-Hôpitaux de Paris, 51, Avenue du Maréchal de Lattre de Tassigny, 94010 Créteil Cedex, France; 2 Groupe de Recherche Clinique CARMAS, Faculté de Santé, Université Paris Est-Créteil, 94010 Créteil Cedex, France; 3 INSERM U955, Institut Mondor de Recherche Biomédicale, 94010 Créteil Cedex, France; 4 Service de Médecine Intensive-Réanimation, Centre Hospitalier Intercommunal de Créteil, Créteil, France; 5 Service de Réanimation, Centre Hospitalier de La Rochelle, La Rochelle, France; 6 University of São Paulo, São Paulo, Brazil

**Keywords:** ARDS, Electrical impedance tomography, Ventilation, Perfusion, Ventilation/perfusion mismatch, Shunt, Dead space

## Abstract

**Introduction:**

Electrical impedance tomography (EIT) can be used to assess ventilation/perfusion (*V*/*Q*) mismatch within the lungs. Several methods have been proposed, some of them neglecting the absolute value of alveolar ventilation (*V*_A_) and cardiac output (*Q*_C_). Whether this omission results in acceptable bias is unknown.

**Methods:**

Pixel-level *V*/*Q* maps of 25 ARDS patients were computed once considering (absolute *V*/*Q* map) and once neglecting (relative *V*/*Q* map) the value of *Q*_C_ and *V*_A_. Previously published indices of *V*/*Q* mismatch were computed using absolute *V*/*Q* maps and relative *V*/*Q* maps. Indices computed with relative *V*/*Q* maps were compared to their counterparts computed using absolute *V*/*Q* maps.

**Results:**

Among 21 patients with ratio of alveolar ventilation to cardiac output (*V*_A_/*Q*_C_) > 1, relative shunt fraction was significantly higher than absolute shunt fraction [37% (24–66) vs 19% (11–46), respectively, *p* < 0.001], while relative dead space fraction was significantly lower than absolute dead space fraction [40% (22–49) vs 58% (46–84), respectively, *p* < 0.001]. Relative wasted ventilation was significantly lower than the absolute wasted ventilation [16% (11–27) vs 29% (19–35), respectively, *p* < 0.001], while relative wasted perfusion was significantly higher than absolute wasted perfusion [18% (11–23) vs 11% (7–19), respectively, *p* < 0.001]. The opposite findings were retrieved in the four patients with *V*_A_/*Q*_C_ < 1.

**Conclusion:**

Neglecting cardiac output and alveolar ventilation when assessing *V*/*Q* mismatch indices using EIT in ARDS patients results in significant bias, whose direction depends on the *V*_A_/*Q*_C_ ratio value.

**Supplementary Information:**

The online version contains supplementary material available at 10.1186/s13054-023-04467-w.

## Introduction

Acute respiratory distress syndrome (ARDS) is associated with lung ventilation and perfusion mismatch, whose severity is associated with poor prognosis [1]. Electrical impedance tomography (EIT) can be used to assess distribution of both ventilation and perfusion within the lungs [2]. Several methods for calculating pixel-level *V*/*Q* ratios have been reported [2, 3]. We proposed a method based on the calculation of the ventilation and perfusion received by each pixel, allowing a pixel by pixel calculation of the "absolute" pixel-level *V*/*Q* ratio [4]. More recently, Pavlovski et al. reported a method also based on a pixel-by-pixel analysis, but without considering actual cardiac output (*Q*_C_) and alveolar ventilation (*V*_A_) in the calculation of the pixel-level *V*/*Q* ratio [5], which were instead calculated from the relative participation of each pixel in ventilation and perfusion, implicitly considering that cardiac output and alveolar ventilation are equal. Although this method has the advantage of allowing estimation of pixel-level *V*/*Q* ratios with EIT data alone, whether it results in acceptable bias is unknown.

Our objective was to evaluate the impact of neglecting cardiac output and alveolar ventilation when assessing ventilation/perfusion mismatch indices using EIT.

## Methods

This is an ancillary and retrospective analysis of data from patients included in a prospective single-center study (ethic agreement: CPP-66/17) investigating the impact of PEEP variations and prone position on ventilation and perfusion distributions [4]. Recordings made with a PEEP level of 12 cmH_2_O in the supine position were selected for this study.

Patients were intubated, sedated, paralyzed, ventilated in volume control mode with a tidal volume between 6 and 8 mL/kg of predicted body weight (PBW). Ventilation and perfusion distributions were studied using the Enlight 1800 (TIMPEL SA, São Paulo, Brazil). Cardiac output (*Q*_C_) was measured using the Vigileo device (Edwards Lifesciences, Irvine, CA), in hemodynamically stabilized patients. Alveolar ventilation (*V*_A_) was calculated as 70% of the expiratory minute ventilation measured on the mechanical ventilator (in order to remove ventilation of anatomical and instrumental dead space). The ventilation map, which divided the thoracic field into 1024 pixels (32 × 32), was generated using a dedicated software (Timpel offline analysis, TIMPEL SA, São Paulo, Brazil). This map indicated, for each pixel, the fraction of the total alveolar ventilation directed to that pixel. The perfusion map was created as previously described [6]. The perfusion map also included 1024 pixels (32 × 32), corresponding one by one to the pixels of the ventilation map, and indicated, for each pixel, the fraction of the total pulmonary perfusion directed to that pixel.

The pixel-level *V*/*Q* ratio was then calculated, for each pixel, by two distinct methods: without taking into account (“relative” pixel-level *V*/*Q*) and with taking into account (“absolute” pixel-level *V*/*Q*) cardiac output and alveolar ventilation:

- “relative” pixel-level *V*/*Q* ${\left(\frac{V}{Q}\right)}_{\mathrm{PIXEL},\mathrm{REL}}$
  - The percentage of total ventilation captured by pixel *i* (*V*%,*i*) was divided by the percentage of total perfusion captured by the same pixel (*Q*%,*i*), as in [5].
    $${\left(\frac{V}{Q}\right)}_{\mathrm{PIXEL},\mathrm{ REL},i}=\frac{{V}_{{\%},i}}{{Q}_{{\%},i}}:$$
- “absolute” pixel-level *V*/*Q* ${\left(\frac{V}{Q}\right)}_{\mathrm{PIXEL},\mathrm{ ABS}}$
  - First, the percentage of total ventilation captured by pixel *i* (*V*%,*i*) was multiplied by the total alveolar ventilation, in order to calculate the absolute alveolar ventilation of this pixel
  - Second, the percentage of total perfusion captured by pixel *i* (*Q*%,*i*) was multiplied by the cardiac output, in order to calculate the absolute perfusion of this pixel
  - Third, the absolute pixel-level *V*/*Q* ratio was calculated by dividing the absolute ventilation by the absolute perfusion of pixel *i*:
    $${\left(\frac{V}{Q}\right)}_{\mathrm{PIXEL},\mathrm{ ABS}, i} =\frac{{V}_{{\%},\mathrm{i}}*{V}_{\mathrm{A}}}{{Q}_{{\%},\mathrm{i}}* {Q}_{\mathrm{c}}}$$

To estimate the potential impact of neglecting the alveolar ventilation and cardiac output on the estimation of ventilation/perfusion mismatch, the following variables were computed both using ${\left(\frac{V}{Q}\right)}_{\mathrm{PIXEL},\mathrm{ ABS}}$ and ${\left(\frac{V}{Q}\right)}_{\mathrm{PIXEL},\mathrm{ REL}}$:

- Distribution of pixels among five different classes of ventilation/perfusion match: shunt pixels (pixel-level *V*/*Q* ratio ≤ 0.1); pixels with low *V*/*Q* ratio (pixel-level *V*/*Q* ratio 0.1–0.8); pixels with normal *V*/*Q* ratio (pixel-level *V*/*Q* ratio 0.8–1.25); pixels with high *V*/*Q* ratio (*V*/*Q* ratio 1.25–10); dead space pixels (pixel-level *V*/*Q* ratio ≥ 10). These definitions of *V*/*Q* unmatching were defined by analogy to [5].
- Shunt fraction [4]: the fraction of cardiac output reaching pixels with shunt or low *V*/*Q* ratio
- Dead space fraction [4]: the fraction of alveolar ventilation reaching pixels with dead space or high *V*/*Q* ratio
- Wasted ventilation, as defined by [5]
- Wasted perfusion, as defined by [5]

Value of each variable computed using ${\left(\frac{V}{Q}\right)}_{\mathrm{PIXEL},\mathrm{ ABS}}$(absolute shunt fraction, absolute dead space fraction, absolute wasted ventilation and absolute wasted perfusion) was compared to the value computed using ${\left(\frac{V}{Q}\right)}_{\mathrm{PIXEL},\mathrm{ REL}}$ (relative shunt fraction, relative dead space fraction, relative wasted ventilation and relative wasted perfusion) by Wilcoxon paired test. Since the direction of the changes in ventilation/perfusion mismatch indices between the two methods (with and without consideration of the cardiac output and alveolar ventilation) is assumed to be opposite between patients with alveolar ventilation superior to cardiac output and those with alveolar ventilation inferior to cardiac output, we planned to analyze these two groups separately. To test the robustness of our results with respect to the threshold defining a normal ventilation/perfusion ratio, we performed a sensitivity analysis, considering a normal ventilation/perfusion ratio between 0.5 and 2. To determine whether our results were robust to the definition of alveolar ventilation, we repeated the analyses by calculating anatomic dead space using two other formulas [7, 8]. More details about definitions and methods are available in the online supplement. Results are reported as median and interquartile range. Statistical analysis was made with GraphPad Prism version 9.4.1 (GraphPad Software, LLC).

## Results

Twenty-five ARDS patients were included in the analysis. Detailed patient characteristics are presented in the online supplement (Additional file 1: Table S1). Twenty-one patients exhibited an alveolar ventilation superior to their cardiac output and four an alveolar ventilation inferior to the cardiac output.

**Fig. 1:**
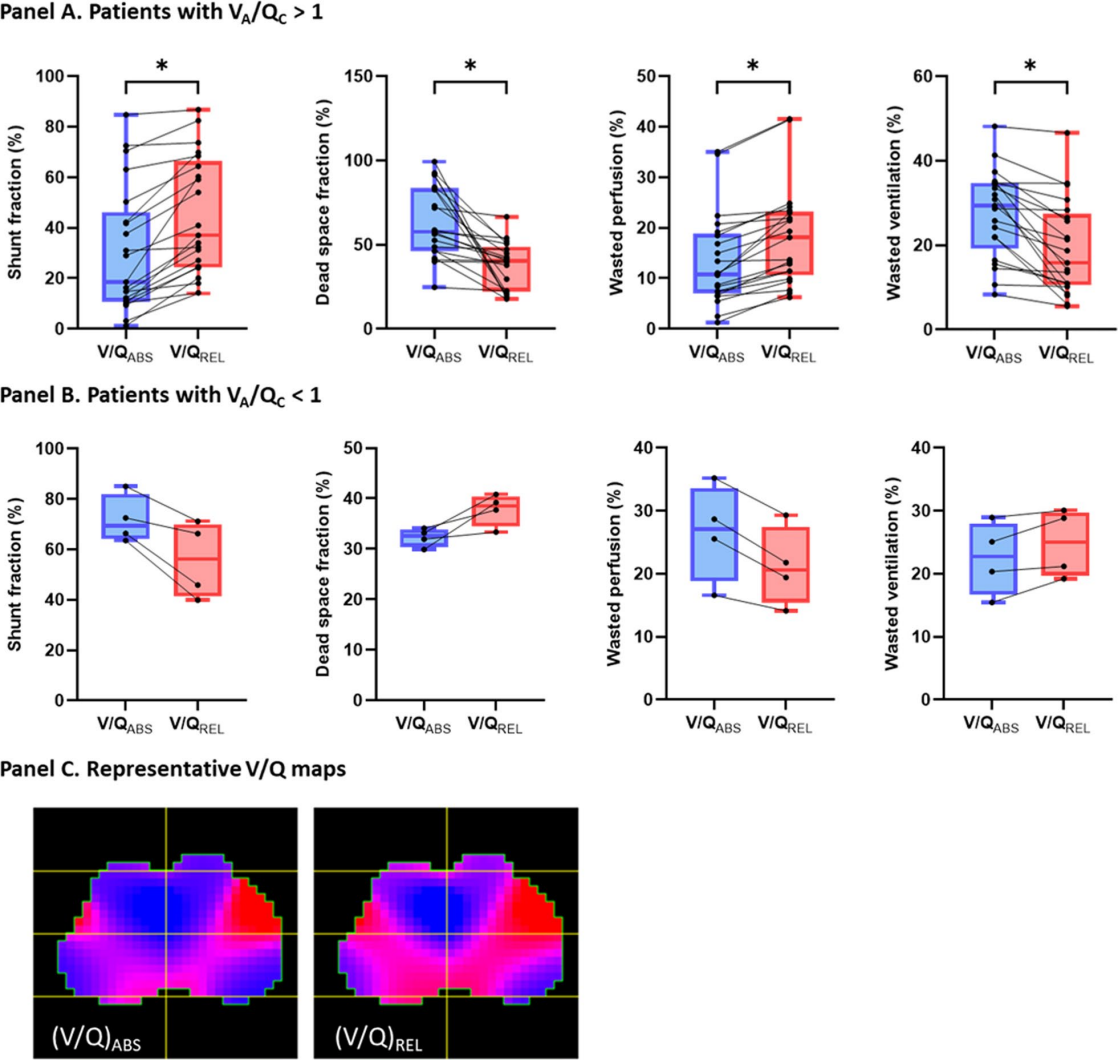
Differences in ventilation/perfusion matching indices depending on the accounting of cardiac output and alveolar ventilation. **A** Patients with *V*_A_/*Q*_C_ > 1. From left to right: shunt fraction, dead space fraction, wasted perfusion, wasted ventilation. Blue boxes represent absolute indices. Red boxes represent relative indices. Individual patient data are represented by black dots. * denotes *p* < 0.0001. **B** Patients with *V*_A_/*Q*_C_ < 1. From left to right: shunt fraction, dead space fraction, wasted perfusion, wasted ventilation. Blue boxes represent absolute indices. Red boxes represent relative indices. Individual patient data are represented by black dots. **C** *V*/*Q* maps of a representative patient. Left: absolute *V*/*Q* map. Right: relative *V*/*Q* map. Blue pixels correspond to high *V*/*Q* and dead space pixels. Red pixels correspond to low *V*/*Q* and shunt pixels. Pink pixels correspond to pixels with normal *V*/*Q*

Among the 21 patients with alveolar ventilation superior to cardiac output, the ${\left(\frac{V}{Q}\right)}_{\mathrm{PIXELREL}}$ systematically classified more pixels in low *V*/*Q* and less pixels in high *V*/*Q* compared to ${\left(\frac{V}{Q}\right)}_{\mathrm{PIXELABS}}$ (Table 1). Thus, relative shunt fraction was significantly higher than absolute shunt fraction [37% (24–66) vs 19% (11–46), respectively, *p* < 0.001; Bland–Altman comparison: bias 14%, LAL − 6%, UAL 33%], while relative dead space fraction was significantly lower than absolute dead space fraction [40% (22–49) vs 58% (46–84), respectively, *p* < 0.001; Bland–Altman comparison: bias − 27%, LAL − 79%, UAL 25%] (Fig. 1). Relative wasted ventilation was significantly lower than the absolute wasted ventilation [16% (11–27) vs 29% (19–35), respectively, *p* < 0.001; Bland–Altman comparison: bias − 8%, LAL − 23%, UAL 6%], while relative wasted perfusion was also significantly higher than absolute wasted perfusion [18% (11–23) vs 11% (7–19), respectively, *p* < 0.001; Bland–Altman comparison: bias 5%, LAL − 1%, UAL 11%]. Bland–Altman plots are available in the supplement (Additional file 1: Figure S1).

**Table 1:** Comparison of *V*/*Q* mismatch indices between absolute and relative *V*/*Q*

	All patients (*n* = 25)			Patients with *V*_A_/*Q*_C_ > 1 (*n* = 21)			Patients with *V*_A_/*Q*_C_ < 1 (*n* = 4)		
	Absolute *V*/*Q*	Relative *V*/*Q*	*p*	Absolute *V*/*Q*	Relative *V*/*Q*	*p*	Absolute *V*/*Q*	Relative *V*/*Q*	*p*
Alveolar ventilation (L min^−1^)	7.9 (7.5; 9.3)			7.9 (7.6; 9.3)		NA	7.7 (6.8; 9.6)		NA
Cardiac output (L min^−1^)	6.3 (5.0; 8.5)			5.9 (4.9; 7.7)		NA	9.0 (8.6; 11.4)		NA
*Pixels with*									
Shunt (*V*/*Q* ≤ 0.1) (%)	11 (7; 15)	11 (7; 15)	1	11 (7; 15)	11 (7; 15)	1	16 (5; 30)	16 (5; 30)	NA
Low *V*/*Q* (0.1 < *V*/*Q* < 0.8) (%)	19 (6; 28.0)	23 (18; 36)	0.002	10 (5; 25)	23 (17; 35)	< 0.0001	40 (31; 50)	22 (16; 26)	NA
Normal *V*/*Q* (0.8 ≤ *V*/*Q* ≤ 1.25) (%)	17 (11; 22)	24 (15; 45)	0.03	17 (11; 28)	28 (14; 48)	0.19	17 (9; 18)	85 (48; 109)	NA
High *V*/*Q* (1.25 < *V*/*Q* < 10) (%)	27 (12; 48)	15 (12; 22)	< 0.001	33 (25; 54)	15 (12; 23)	< 0.0001	9 (5; 12)	12 (8; 16)	NA
Dead space (*V*/*Q* ≥ 10) (%)	17 (7; 24)	17 (7; 24)	1	17 (6; 22)	17 (6; 22)	1	21 (12; 28)	21 (12; 28)	NA
Shunt fraction (%)	31 (12; 65)	41 (26; 67)	0.006	19 (11; 46)	37 (24; 66)	< 0.0001	70 (64; 82)	56 (41; 70)	NA
Dead space fraction (%)	56 (41–83)	40 (23; 67)	0.0001	58 (46; 84)	40 (22; 49)	< 0.0001	33 (30; 34)	39 (34; 40)	NA
Wasted ventilation (%)	29 (18; 35)	19 (11; 29)	< 0.001	29 (19; 35)	16 (11; 27)	< 0.0001	23 (17; 28)	25 (20; 30)	NA
Wasted perfusion (%)	13 (7; 22)	19 (11; 23)	0.005	11 (7; 19)	18 (11; 23)	< 0.0001	27 (19; 34)	21 (15; 27)	NA

*V*_A_, alveolar ventilation; *Q*_C_, cardiac output; *V*/*Q*, ventilation to perfusion ratio at the pixel level

The opposite findings were retrieved in the four patients with alveolar ventilation inferior to cardiac output (Table 1 and Fig. 1), but the low sample size precluded any statistical analysis.

Difference between relative and absolute wasted ventilation on the one hand, and relative and absolute wasted perfusion on the other hand, were both correlated with the ratio of alveolar ventilation to cardiac output (Additional file 1: Figure S2 and S3).

Sensitivity analysis with a definition of normal pixel-level *V*/*Q* ratio between 0.5 and 2 yielded similar results (Additional file 1: Table S2). Similarly, analysis considering other formulas for calculating alveolar ventilation yielded similar results (Additional file 1: Tables S3 and S4).

## Discussion

The main finding of our study is that the relative and absolute pixel-level *V*/*Q* calculations resulted in significantly different ventilation/perfusion mismatch assessments.

Assessing ventilation/perfusion mismatch may help customizing mechanical ventilation. However, the ventilator settings themselves, in particular the level of PEEP, may alter the cardiac output [9] in a way that is not predictable [10]. Thus, even in a single patient, comparing ventilation/perfusion mismatch indices across multiple ventilatory strategies, neglecting the actual alveolar ventilation to cardiac output ratio, seems hazardous.

Our study has several limitations:

1. The sample size is modest. Nevertheless, measured cardiac output and minute ventilation were consistent with previous reports [11, 12].
2. Alveolar ventilation was not measured but calculated as a fraction of the minute ventilation. However, repeating the analyses with different formulas for estimating the anatomical dead space [7, 8, 13] yielded consistent results.
3. Patients were assessed at a single PEEP level. However, the level chosen (12 cm H_2_O) was intermediate in order not to promote extreme values of alveolar ventilation to cardiac output ratios.
4. The thresholds defining pixel-level *V*/*Q* mismatch were arbitrary. We chose these thresholds to be consistent with the existing literature. Sensitivity analysis using larger thresholds to define normal *V*/*Q* yielded similar results. Finally, wasted ventilation and wasted perfusion are insensitive to the thresholds defining abnormal ventilation/perfusion ratios, allowing this limitation to be overcome.
5. There is a theoretical risk of concluding that the ventilation/perfusion ratio is normal at the pixel level, while ventilation/perfusion abnormalities exist at the alveolar level, with opposite anomalies canceling each other out. However, given the spatial resolution of EIT (depending on the patient, between 300 and 500 pixels represent the lung), this risk seems minimal.

In conclusion, neglecting cardiac output and alveolar ventilation when assessing *V*/*Q* mismatch indices using EIT in ARDS patients results in significant bias, whose direction depends on the ratio of alveolar ventilation to cardiac output.

## Supplementary Information

**Additional file 1**. **Supplementary appendix**: detailed methods and supplemental results.

## Data Availability

The datasets used and/or analyzed during the current study are available from the corresponding author on reasonable request.

## Abbreviations

ARDS: Acute respiratory distress syndrome
EIT: Electrical impedance tomography
PEEP: Positive end-expiratory pressure
*Q*_C_: Cardiac output
*V*_A_: Alveolar ventilation
*V*/*Q*: Ventilation to perfusion ratio
