# Supplementary material for: Impact of cardiac output and alveolar ventilation in estimating ventilation/perfusion mismatch in ARDS using electrical impedance tomography
Source: Crit Care. 2023 May 8;27:176. doi: 10.1186/s13054-023-04467-w (PMC10165791; doi:10.1186/s13054-023-04467-w)
Supplement: Supplementary file 1 — Additional file 1. Supplementary appendix: detailed methods and supplemental results. [file 13054_2023_4467_MOESM1_ESM.docx]

**Impact of cardiac output and alveolar ventilation in estimating ventilation/perfusion mismatch in ARDS**

Samuel Tuffet^1,2,3^, MD; Tommaso Maraffi^2,3,4^, MD; Matthieu Lacheny^1^; François Perier^5^, MD; Anne-Fleur Haudebourg^1,2,3^, MD; Mohamed Ahmed Boujelben^1,2,3^, MD; Glasiele Alcala^6^, Armand Mekontso-Dessap^1,2,3^, MD, PhD; and Guillaume Carteaux^1,2,3^, MD, PhD

Table of content

[Detailed methods 1](#_Toc123642460)

[Ventilation and perfusion maps 1](#_Toc123642461)

[V/Q ratios 2](#_Toc123642462)

[Distribution of ventilation and perfusion 3](#_Toc123642463)

[Shunt fraction and dead space fraction 4](#_Toc123642464)

[Wasted ventilation and wasted perfusion 4](#_Toc123642465)

[Statistical analysis 5](#_Toc123642466)

[Supplemental Figures and Table 6](#_Toc123642467)

[Table S1. Patients’ characteristics 6](#_Toc123642468)

[Figure S1. Bland-Altman plots representing difference between absolute and relative shunt fraction, dead space fraction, wasted ventilation and wasted perfusion among patients with V_A_/Q_C_ > 1 7](#_Toc123642469)

[Figure S2. Correlation between relative differences in wasted ventilation and V_A_/Q_C_ 8](#_Toc123642470)

[Figure S3. Correlation between relative differences in wasted perfusion and V_A_/Q_C_ 8](#_Toc123642471)

[References 8](#_Toc123642472)

# Detailed methods

## Ventilation and perfusion maps

This is an analysis of data from patients included in a clinical study (ethic agreement: CPP-66/17) investigating the impact of variations of PEEP and prone position on the distribution of ventilation and perfusion. Patients were intubated, sedated, paralyzed, and ventilated in volume control mode with a tidal volume between 6 and 8 mL.kg^-1^ of predicted body weight (PBW). Cardiac output was monitored using the Vigileo device (Edwards Lifesciences, Irvine, CA). Only recordings made at PEEP 12 cmH_2_O and in the supine position were retained for this study.

Ventilation and perfusion distribution were studied using the Enlight 1800 (TIMPEL SA, São Paulo, Brazil). A bolus of 10 mL of 7.5% hypertonic saline was used to determine perfusion distribution during a respiratory pause performed at a pressure level corresponding to the mean airway pressure found during tidal ventilation. The pixel level ventilation map was calculated from a dedicated software (Timpel offline analysis, TIMPEL SA, São Paulo, Brazil) based on the tidal impedance variation of each pixel. Five respiratory cycles (the five last cycles before tele-expiratory occlusion was performed to study perfusion) were included to compute each ventilation map.

The perfusion map was performed as previously described (1). The relative perfusion of each pixel was calculated by performing a linear regression on the impedance curve for each pixel. The start of the regression period was one heartbeat after the start of the global impedance decrease. The end of the regression period was the minimum of the global impedance. The perfusion map was created from the regression slope calculated for each pixel.

## V/Q ratios

A pixel was considered ventilated if its impedance variation on the ventilation map was at least 10% of the impedance variation of the most ventilated pixel. Similarly, a pixel was considered perfused if its impedance variation on the perfusion map was at least 10% of the impedance variation of the most perfused pixel (2). The V/Q ratios for each pixel were then calculated in two ways:

- “relative” V/Q $\left( \frac{V}{Q} \right)_{REL}$: percentage of total ventilation arriving at pixel i (V%,i) was divided by percentage of total perfusion arriving at that pixel (Q%,i), as in (2):

$$\left( \frac{V}{Q} \right)_{REL,i}=\frac{V_{\%,i}}{Q_{\%,i}}$$

- “absolute” V/Q $\left( \frac{V}{Q} \right)_{\mathrm{ABS}}$
  - percentage of total ventilation arriving at pixel i (V%,i) was multiplied by the total alveolar ventilation, allowing to calculate the absolute alveolar ventilation of this pixel
  - percentage of total perfusion arriving at pixel i (Q%,i) was multiplied by the cardiac output, allowing calculation of the absolute perfusion of that pixel
  - the absolute ventilation of each pixel was divided by the absolute perfusion of that pixel to calculate the absolute V/Q ratio

$\left( \frac{V}{Q} \right)_{ABS, i}$ $=\frac{V_{\%,i}*V_{A}}{Q_{\%,i}* Q_{c}}$

Pixels were classified according to their V/Q ratio as described in (2)

- Non-ventilated, perfused pixel (V/Q ratio  ≤ 0.1);
- Pixels with low V/Q ratio (V/Q ratio 0.1–0.8)
- Pixels with normal V/Q ratio (V/Q ratio 0.8–1.25)
- Pixels with high V/Q ratio (V/Q ratio 1.25–10)
- Non-perfused, ventilated pixels (V/Q ratio  ≥ 10).

## Rationale for alveolar ventilation calculation

Our aim was to consider total alveolar ventilation (including alveolar dead space). Therefore, we have to remove from the minute ventilation the ventilation related to the anatomical and instrumental dead space. Since we did not have volumetric capnography data to measure this directly, we relied on formulas to predict anatomical and instrumental dead space in ventilated patients. The methods that provides the best estimates (narrowest limits of agreement) are the followings (3):

- Nielsen's method (4), which defines that anatomical dead space in mL is equal to predicted body weight (IBW) in pounds. This corresponds to estimate that the anatomical dead space represents 36.7% of the minute ventilation in a patient ventilated at 6 mL/kg of IBW. As illustrated in (3), this method most often overestimates the anatomical dead space.
- The Suwa method (5), which defines that anatomical dead space in mL is equal to 0.66* IBW in pounds. This corresponds to an estimate that the anatomical dead space is 24.2% of the minute ventilation in a patient ventilated at 6 mL/kg IBW. As shown in (3), this method most often underestimates the anatomical dead space.

Based on these data, we chose to define the anatomical (and instrumental) dead space as being equal to 30% of the minute ventilation. Thus, alveolar ventilation was equal to 70% of minute ventilation.

## Distribution of ventilation and perfusion

The percentage of ventilation reaching each V/Q class (i.e. Non-ventilated, perfused; low V/Q ratio; normal V/Q ratio; high V/Q ratio; non-perfused, ventilated) was computed by adding the percentages of ventilation received by each pixel of that V/Q class.

The percentage of cardiac output reaching each V/Q class (i.e. Non-ventilated, perfused; low V/Q ratio; normal V/Q ratio; high V/Q ratio; non-perfused, ventilated) was computed by adding the percentages of perfusion received by each pixel of that V/Q class.

Each analysis was done twice: once with $\left( \frac{V}{Q} \right)_{\mathrm{ABS}}$and once with $\left( \frac{V}{Q} \right)_{REL}$.

## Shunt fraction and dead space fraction

Similarly to (6) :

- Shunt fraction was defined as the percentage of cardiac output reaching pixels with shunt (non-ventilated, perfused pixels) or low V/Q pixels.
- Dead space fraction was defined as the percentage of alveolar ventilation reaching pixels with high V/Q ratios or dead space pixels (ventilated, non-perfused pixels)

## Wasted ventilation and wasted perfusion

As described in (2), the decimal logarithm of the V/Q ratio of each pixel was computed, and then rounded to its first decimal on a scale between − 1 and 1.

Wasted ventilation, wasted perfusion, distribution of ventilation and perfusion using $\left( \frac{V}{Q} \right)_{REL}$and $\left( \frac{V}{Q} \right)_{\mathrm{ABS}}$were computed as follows :

$$Wasted ventilation= \sum_{i=1}^{n} \left( {\log\left( \dot{\frac{\dot{V}}{Q}} \right)}_{i}*{Vt}_{i} \right)$$

where n is the number of pixels in the functional EIT image, including only units with V/Q ratio >1

$$Wasted perfusion= \sum_{i=1}^{n} \left( {\log\left( \dot{\frac{\dot{V}}{Q}} \right)}_{i}*Q_{i} \right)$$

where n is the number of pixels in the functional EIT image, including only units V/Q ratio <1

For both equations, the absolute value of the logarithm was taken into account.

## Statistical analysis

Each of the analysis was done twice for each patient: once with $\left( \frac{V}{Q} \right)_{\mathrm{ABS}}$and once with $\left( \frac{V}{Q} \right)_{REL}$. Using both Wilcoxon paired test and Bland-Altman comparison method, we compared absolute with relative wasted ventilation, absolute with relative wasted perfusion, absolute with relative dead space fraction, and absolute with relative shunt fraction

Relative difference in wasted ventilation was computed as

$$Relative difference in wasted ventilation = \frac{Relative wasted ventilation-Absolute wasted ventilation}{Absolute wasted ventilation}$$

Relative difference in wasted perfusion was computed as

$$Relative difference in wasted perfusion = \frac{Relative wasted perfusion-Absolute wasted perfusion}{Absolute wasted perfusion}$$

Correlation between relative difference in wasted ventilation and V_A_/Q_C_, and correlation between relative difference in wasted perfusion and V_A_/Q_C_ were studied using linear regression.

# Supplemental Figures and Table

## Table S1. Patients’ characteristics

| Variable | | All cohort (n=25) |
| --- | --- | --- |
| Age, years | | 59 (51-71) |
| Weight, kg | | 80 (65-95) |
| Height, cm | | 170 (165-175) |
| Body mass index, kg.m^-2^ | | 28,9 (22,4-32,9) |
| Predicted body weight (PBW), kg | | 63 (61-69) |
| SARS-CoV-2 related ARDS, n (%) | | 12 (48%) |
| Ventilatory parameters | |  |
|  | Tidal volume (mL) | 390 (365-432) |
|  | Tidal volume / PBW (mL.kg^-1^) | 6,1 (5,8-6,4) |
|  | Respiratory rate, min^-1^ | 29 (28-31) |
|  | Minute ventilation, L.min^-1^ | 11,3 (10,6-13,3) |
|  | FiO_2_ | 0,7 (0,6-0,8) |
|  | Set PEEP, cmH_2_O | 12 (12-12) |
|  | Total PEEP, cmH_2_O | 13 (13-14) |
|  | Plateau pressure, cmH_2_O | 23 (22-28) |
|  | Respiratory system compliance (mL.cmH_2_O^-1^) | 38 (26-46) |
|  | SpO2 (%) | 97 (95-98) |
|  | EtCO2 (mmHg) | 30 (25-36) |
| Arterial Blood Gases | |  |
|  | pH | 7.30 (7.24-7.38) |
|  | [HCO3-] (mmol.L^-1^) | 24.0 (20.6-25.0) |
|  | PaCO2 (mmHg) | 43 (40-47) |
|  | PaO2 (mmHg) | 84 (76-106) |
|  | SaO2 (%) | 95 (93-98) |
|  | PaO2/FiO2 (mmHg) | 140 (111-182) |
| Hemodynamic parameters | |  |
|  | Use of vasoconstrictors, n (%) | 21 (84%) |
|  | Systolic arterial pressure (mmHg) | 122 (114-135) |
|  | Diastolic arterial pressure (mmHg) | 56 (53-65) |
|  | Mean arterial pressure (mmHg) | 78 (71-84) |
|  | Heart rate (min^-1^) | 97 (73-110) |
|  | Stroke volume (mL) | 71 (49-94) |
|  | Cardiac output (L.min^-1^) | 6.3 (5-8.5) |

PBW : predicted body weight ; FiO_2 :_ inspired fraction of oxygen ; PEEP : positive end-expiratory pressure ; SpO2 : transcutaneous oxygen saturation ; EtCO2 : end-tidal carbon dioxide pressure ; PaCO2 : arterial carbon dioxide pressure ; PaO2 : arterial oxygen pressure ; SaO2 : arterial oxygen saturation.

## Figure S1. Bland-Altman plots representing difference between absolute and relative shunt fraction, dead space fraction, wasted ventilation and wasted perfusion among patients with V_A_/Q_C_ > 1


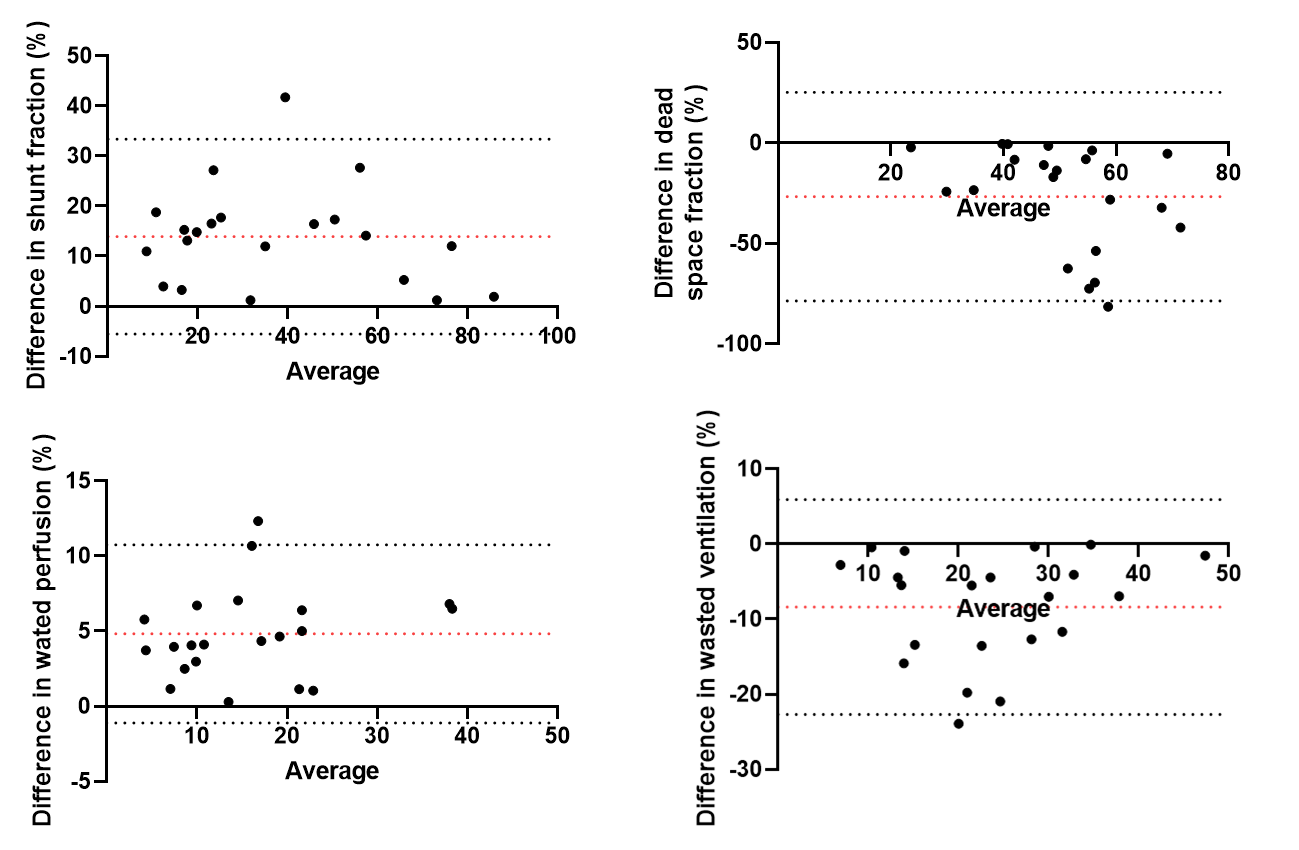


Top left : Bland-Altman plot of differences between relative and absolute shunt fraction ; Top right : Bland-Altman plot of differences between relative and absolute dead space fraction ; Bottom left : Bland-Altman plot of differences between relative and absolute wasted perfusion ; Bottom right : Bland-Altman plot of differences between relative and absolute wasted ventilation. Red dashed line represents bias. Black dashed lines represent lower and upper agreement limits

## Figure S2. Correlation between relative differences in wasted ventilation and V_A_/Q_C_

## Figure S3. Correlation between relative differences in wasted perfusion and V_A_/Q_C_

#

# Table S2. Comparison of V/Q mismatch indices between absolute and relative V/Q. Other definition of normal pixel-level V/Q

Normal pixel-level V/Q is here defined by 0.5<V/Q<2

|  | | All patients (n=25) | | | Patients with V_A_/Q_C_ >1 (n=21) | | | Patients with V_A_/Q_C_ <1 (n=4) | | |
| --- | --- | --- | --- | --- | --- | --- | --- | --- | --- | --- |
|  | | **Absolute V/Q** | **Relative V/Q** | **p** | **Absolute V/Q** | **Relative V/Q** | **p** | **Absolute V/Q** | **Relative V/Q** | **p** |
| Alveolar ventilation (L.min^-1^) | | 7.9 (7.5 ; 9.3) |  |  | 7.9 (7.6 ; 9.3) |  | NA | 7.7 (6.8 ; 9.6) |  | NA |
| Cardiac output (L.min^-1^) | | 6.3 (5.0 ; 8.5) |  |  | 5.9 (4.9 ; 7.7) |  | NA | 9.0 (8.6 ; 11.4) |  | NA |
| Pixels with | |  |  |  |  |  |  |  |  |  |
|  | Shunt (V/Q≤0.1) (%) | 11 (7 ; 15) | 11 (7  ; 15) | 1 | 11 (7 ; 15) | 11 (7 ; 15) | 1 | 16 (5 ; 30) | 16 (5 ; 30) | NA |
|  | Low V/Q (0.1<V/Q<0.5) (%) | 2 (0; 10) | 5 (2 ; 11) | 0.02 | 1 (0 ; 5) | 4 (2 ; 13) | <0.0001 | 11 (7 ; 18) | 5 (4 ; 10) | NA |
|  | Normal V/Q (0.5≤V/Q≤2) (%) | 51 (42 ; 68) | 58 (45 ; 74) | 0.008 | 54 (44 ; 70) | 61 (45 ; 75) | 0.04 | 46 (38 ; 58) | 52 (44; 60) | NA |
|  | High V/Q (2<V/Q<10) (%) | 12 (4; 17) | 4 (2; 6) | <0.0001 | 13 (5; 20) | 4 (2; 7) | <0.0001 | 3 (2; 4) | 5 (3; 6) | NA |
|  | Dead space (V/Q≥10) (%) | 17 (7 ; 24) | 17 (7 ; 24) | 1 | 17 (6 ; 22) | 17 (6 ; 22) | 1 | 21 (12 ; 28) | 21 (12 ; 28) | NA |
| Shunt Fraction (%) | | 9 (5; 22) | 17 (7; 25) | 0.01 | 7 (4; 18) | 12 (7; 25) | <0.0001 | 30 (13; 36) | 20 (10; 26) | NA |
| Dead space fraction (%) | | 32 (18; 44) | 20 (9; 31) | 0.0001 | 33 (18; 45) | 17 (8; 31) | <0.0001 | 24 (18; 31) | 26 (21; 32) | NA |
| Wasted ventilation (%) | | 29 (18 ; 35) | 19 (11 ; 29) | <0.0001 | 29 (19 ; 35) | 16 (11 ; 27) | <0.0001 | 23 (17 ; 28) | 25 (20 ; 30) | NA |
| Wasted perfusion (%) | | 13 (7 ; 22) | 19 (11 ; 23) | 0.005 | 11 (7 ; 19) | 18 (11 ; 23) | <0.0001 | 27 (19 ; 34) | 21 (15 ; 27) | NA |

V_A_ : alveolar ventilation. Q_C_ : cardiac output. V/Q : ventilation to perfusion ratio at the pixel level.

# Table S3. Comparison of V/Q mismatch indices between absolute and relative V/Q. Suwa method for anatomical dead space estimation

Calculation of anatomical dead space was based on Suwa formula (5)

|  | | All patients (n=25) | | | Patients with V_A_/Q_C_ >1 (n=22) | | | Patients with V_A_/Q_C_ <1 (n=3) | | |
| --- | --- | --- | --- | --- | --- | --- | --- | --- | --- | --- |
|  | | **Absolute V/Q** | **Relative V/Q** | **p** | **Absolute V/Q** | **Relative V/Q** | **p** | **Absolute V/Q** | **Relative V/Q** | **p** |
| Alveolar ventilation (L.min^-1^) | | 8.6 (8; 9.9) |  |  | 8.7 (8.2; 9.8) |  |  | 7.6 (7.3; 11) |  |  |
| Cardiac output (L.min^-1^) | | 6.3 (5; 8.5) |  |  | 6.1 (4.9; 7.9) |  |  | 9.1 (8.5; 12.1) |  |  |
| Pixels with | |  |  |  |  |  |  |  |  |  |
|  | Shunt (V/Q≤0.1) (%) | 11 (7; 15) | 11 (7; 15) | 1 | 11 (6; 15) | 11 (6; 15) | 1 | 23 (8; 32) | 23 (8; 32) | NA |
|  | Low V/Q (0.1<V/Q<0.8) (%) | 15 (4; 25) | 23 (18; 36) | <0.0001 | 8 (4; 23) | 23 (18; 36) | <0.0001 | 32 (24 ; 47) | 56 (18; 42) | NA |
|  | Normal V/Q (0.8≤V/Q≤1.25) (%) | 17 (9; 23) | 24 (15; 45) | 0.03 | 17 (7; 26) | 26 (14 ; 47) | 0.07 | 20 (11 ; 23) | 22 (15; 28) | NA |
|  | High V/Q (1.25<V/Q<10) (%) | 37 (14; 51) | 12 (12; 22) | <0.0001 | 38 (29; 56) | 15 (12; 23) | <0.0001 | 8 (5; 13) | 12 (7; 17) | NA |
|  | Dead space (V/Q≥10) (%) | 17 (7; 24) | 17 (7; 24) | 1 | 17 (6; 23) | 17 (6; 23) | 1 | 17 (10; 29) | 17 (10; 29) | NA |
| Shunt Fraction (%) | | 28 (10; 60) | 41 (26; 67) | 0.0001 | 21 (10; 50) | 39 (25; 67) | <0.0001 | 57 (55; 80) | 46 (40; 71) | NA |
| Dead space fraction (%) | | 64 (44; 86) | 40 (23; 46) | <0.0001 | 65 (54; 87) | 40 (22; 48) | <0.0001 | 34 (31; 38) | 39 (38; 40) | NA |
| Wasted ventilation (%) | | 30 (20; 39) | 19 (11; 29) | <0.0001 | 31 (21; 39) | 17 (11; 27) | <0.0001 | 26 (16; 31) | 29 (19; 30) | NA |
| Wasted perfusion (%) | | 12 (7; 19) | 19 (11; 23) | <0.0001 | 9 (6; 17) | 16 (11; 23) | <0.0001 | 25 (24; 32) | 22 (19; 29) | NA |

V_A_ : alveolar ventilation. Q_C_ : cardiac output. V/Q : ventilation to perfusion ratio at the pixel level.

# Table S4. Comparison of V/Q mismatch indices between absolute and relative V/Q. Nielsen method for anatomical dead space estimation

Calculation of anatomical dead space was based on Nielsen formula (4)

|  | | All patients (n=25) | | | Patients with V_A_/Q_C_ >1 (n=18) | | | Patients with V_A_/Q_C_ <1 (n=7) | | |
| --- | --- | --- | --- | --- | --- | --- | --- | --- | --- | --- |
|  | | **Absolute V/Q** | **Relative V/Q** | **p** | **Absolute V/Q** | **Relative V/Q** | **p** | **Absolute V/Q** | **Relative V/Q** | **p** |
| Alveolar ventilation (L.min^-1^) | | 7.3 (6.5; 8.3) |  |  | 7.3 (6.8; 8.4) |  |  | 7.3 (6.1; 7.6) |  |  |
| Cardiac output (L.min^-1^) | | 6.3 (5; 8.5) |  |  | 5.3 (4.8; 7.6) |  |  | 8.8 (7.6; 9.1) |  |  |
| Pixels with | |  |  |  |  |  |  |  |  |  |
|  | Shunt (V/Q≤0.1) (%) | 11 (7; 15) | 11 (7; 15) | 1 | 11 (7; 15) | 11 (7; 15) | 1 | 9 (2; 23) | 9 (2; 23) | 1 |
|  | Low V/Q (0.1<V/Q<0.8) (%) | 23 (8; 31) | 23 (18; 36) | 0.04 | 13 (7; 25) | 23 (16; 35) | <0.0001 | 45 (31 ; 49) | 36 (21 ; 42) | 0.03 |
|  | Normal V/Q (0.8≤V/Q≤1.25) (%) | 17 (10 ; 29) | 24 (15; 45) | 0.03 | 17 (10; 33) | 28 (15; 50) | 0.3 | 11 (6; 16) | 22 (11; 28) | 0.06 |
|  | High V/Q (1.25<V/Q<10) (%) | 25 (9; 37) | 15 (12; 22) | 0.004 | 32 (22; 53) | 15 (12; 22) | <0.0001 | 7 (5; 10) | 12 (7; 17) | 0.03 |
|  | Dead space (V/Q≥10) (%) | 17 (7; 24) | 17 (7; 24) | 1 | 17 (6; 21) | 17 (6; 21) | 1 | 25 (10; 33) | 25 (10; 33) | 1 |
| Shunt Fraction (%) | | 37 (15; 74) | 41 (26; 67) | 0.08 | 21 (12; 46) | 37 (24; 64) | <0.0001 | 81 (71; 89) | 66 (40; 74) | 0.02 |
| Dead space fraction (%) | | 55 (36; 72) | 40 (23; 46) | 0.002 | 60 (43; 16) | 40 (22; 50) | <0.0001 | 35 (28; 41) | 40 (38; 41) | 0.08 |
| Wasted ventilation (%) | | 26 (15; 32) | 19 (11; 29) | 0.0006 | 27 (17; 35) | 16 (10; 57) | <0.0001 | 25 (14; 30) | 28 (19; 30) | 0.38 |
| Wasted perfusion (%) | | 15 (9; 26) | 19 (11; 23) | 0.15 | 10 (8; 20) | 18 (10; 23) | <0.0001 | 27 (19; 32) | 22 (14; 23) | 0.02 |

V_A_ : alveolar ventilation. Q_C_ : cardiac output. V/Q : ventilation to perfusion ratio at the pixel level

# References

1. He H, Chi Y, Long Y, Yuan S, Frerichs I, Möller K, et al. Influence of overdistension/recruitment induced by high positive end-expiratory pressure on ventilation–perfusion matching assessed by electrical impedance tomography with saline bolus. Crit Care. 29 sept 2020;24(1):586.

2. Pavlovsky B, Pesenti A, Spinelli E, Scaramuzzo G, Marongiu I, Tagliabue P, et al. Effects of PEEP on regional ventilation-perfusion mismatch in the acute respiratory distress syndrome. Crit Care. 11 juill 2022;26(1):211.

3. Brewer LM, Orr JA, Pace NL. Anatomic Dead Space Cannot Be Predicted by Body Weight. Respir CARE. 2008;53(7).

4. Nielsen L. Assessing Patients’ Respiratory Problems. AJN Am J Nurs. déc 1980;80(12):2192.

5. Suwa K, Bendixen HH. Change in PaCO2 with mechanical dead space during artificial ventilation. J Appl Physiol. avr 1968;24(4):556‑62.

6. Perier F, Tuffet S, Maraffi T, Alcala G, Victor M, Haudebourg AF, et al. Effect of Positive End-Expiratory Pressure and Proning on Ventilation and Perfusion in COVID-19 Acute Respiratory Distress Syndrome. Am J Respir Crit Care Med. 15 déc 2020;202(12):1713‑7.
